# MicroRNAs in Sarcopenia: A Systematic Review

**DOI:** 10.3389/fmed.2020.00180

**Published:** 2020-05-28

**Authors:** Katsunori Yanai, Shohei Kaneko, Hiroki Ishii, Akinori Aomatsu, Kiyonori Ito, Keiji Hirai, Susumu Ookawara, Kenichi Ishibashi, Yoshiyuki Morishita

**Affiliations:** ^1^Division of Nephrology, First Department of Integrated Medicine, Saitama Medical Center, Jichi Medical University, Saitama, Japan; ^2^Division of Intensive Care Unit, First Department of Integrated Medicine, Saitama Medical Center, Jichi Medical University, Saitama, Japan; ^3^Department of Medical Physiology, Meiji Pharmaceutical University, Tokyo, Japan

**Keywords:** sarcopenia, microRNA, myotube, myocyte, myoblast, systematic review

## Abstract

Sarcopenia, which is characterized by the loss of skeletal muscle, has been reported to contribute to development of physical disabilities, various illnesses, and increasing mortality. MicroRNAs (miRNAs) are small non-coding RNAs that inhibit translation of target messenger RNAs. Previous studies have shown that miRNAs play pivotal roles in the development of sarcopenia. Therefore, this systematic review focuses on miRNAs that regulate sarcopenia.

## Introduction

Sarcopenia, defined by the loss of skeletal muscle loss, contributes to developing physical disabilities, various illnesses, and increasing mortality (1, 2). MicroRNAs (miRNAs) have attracted attention as potential biomarkers and targets for specific therapies. MiRNAs are small non-coding RNAs (21–25 bases) that are not translated into proteins but inhibit the function of their target messenger RNAs (mRNAs) by destabilizing them and inhibiting their translation (3, 4). Previous studies have shown that miRNAs play pivotal roles in the development of sarcopenia (1–82). Therefore, this systematic review focuses on miRNAs that regulate sarcopenia.

## Mechanism of the Development of Sarcopenia

Several factors, including chronic inflammation, increased reactive oxidative species, increased fibrosis of muscle, and increased loss of motor neurons, have been reported to contribute to development of sarcopenia by progressing muscle atrophy that results in lower muscle mass (26, 46). These factors have been reported to be tightly controlled by many signaling pathways and effector proteins, including some crosstalk with the protein synthesis pathway (32). Among these signaling pathways, transforming growth factor-β_1_ (TGF-β_1_) is considered as the main signaling molecule in the development of sarcopenia (47). TGF-β_1_ activates many downstream profibrotic signaling molecules, including mothers against decapentaplegic (Smad), extracellular signal-regulated kinase (ERK), mitogen-activated protein kinase (MAPK), c-Jun N-terminal kinase (JNK), and p38, which contribute to increasing transdifferentiation of myoblasts into myofibroblasts, resulting in development of muscle atrophy and fibrosis (47). Chronic inflammation has also been considered to contribute to the development of sarcopenia through the production of numerous proinflammatory cytokines, including tumor necrosis factor-α (TNF-α), interleukin (IL)-6, and IL-1β, which promote muscle catabolism (64).

## Search Method

**Figure 1:**
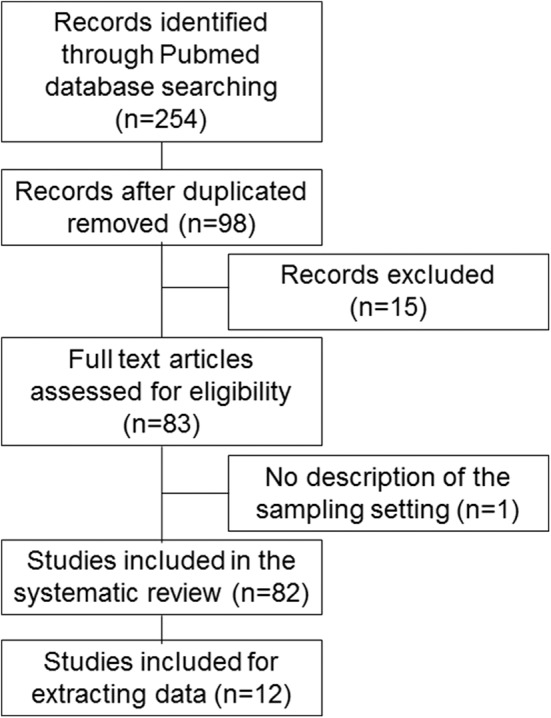
Flow diagram of this study.

We searched for basic and clinical studies published in English in the PubMed database from 2007 to 2019. The literature search was conducted between August 3 and 13, 2019. The following medical subject headings were used: (“microrna AND sarcopenia” [Title/Abstract]), (“mirna AND sarcopenia” [Title/Abstract]), (“microrna AND frail” [Title/Abstract]), (“mirna AND frail” [Title/Abstract]), (“microrna AND frailty” [Title/Abstract]), and (“mirna AND frailty” [Title/Abstract]). The words “frailty” and “frail” were used for this review because they are involved in the condition of sarcopenia. Studies whose titles and abstracts did not meet selection criteria were excluded from this review. The remaining studies were carefully checked for eligibility for inclusion in accordance with Preferred Reporting Items for Systematic Reviews and Meta-Analyses (PRISMA) guidelines [Figure 1; (83)]. The studies were included if (1) they reported the utility of miRNAs as potential biomarkers or targets for specific therapies of sarcopenia; and (2) they were published as full-text journal articles in English. Exclusion criteria were as follows: (1) they did not discuss specific miRNAs in sarcopenia; and (2) they included no description of sample settings. We could not perform a meta-analysis because the number of studies reporting miRNAs for sarcopenia was small, so statistical power would have been low.

## Results

### Search Results

A flow diagram of this study is shown in Figure 1 and Supplemental Table 1. Computer and manual searches identified 254 potentially suitable publications. After the removal of duplicates, the titles and abstracts of 98 remaining papers were screened. Of these, 15 publications were excluded because they did not describe specific miRNAs in sarcopenia, and one publication was excluded because it lacked a description of the sample setting; 82 studies were included in the final systematic review (1–82), and 12 studies were included for extracting data (1, 5, 6, 16, 28, 38, 40, 42, 60, 64, 71, 82).

### MicroRNAs in Sarcopenia

Many studies investigated expression changes of miRNAs in muscles and/or blood (serum or plasma) of patients with sarcopenia (Table 1) and/or animal models of sarcopenia [Table 2; (1, 5, 16, 28, 38, 40, 42, 60, 64, 71, 82)]. Several studies investigated the effects of modulating miRNA expression on phenotypic changes using cultured muscle cells *in vitro* and rodent models of sarcopenia *in vivo* [Table 3; (1, 6, 42, 60, 71)].

**Table 1:** Associations between miRNA expression levels and sarcopenia in patients.

**MiRNA**	**Samples**	**Expression change**	**Details**	**Target mRNA**	**References**
MiRNA-10a-3p	Plasma	↑	The expression level was associated with muscle weight loss, grip strength, self-reported exhaustion, time to walk 15 feet, and kilocalories expended per week	No mention	(40)
MiRNA-19a	Muscle	↑	No mention	PRKAA1 and PFKFB3	(82)
MiRNA-21	Serum	↑	The expression level was associated with self-reported exhaustion, time to walk 15 feet, and muscle weight loss	TGF-βR2	(64)
MiRNA-34a	Muscle	↑	No mention	VEGFA	(82)
MiRNA-92a-3p	Plasma	↑	The expression level was associated with muscle weight loss, grip strength, self-reported exhaustion, time to walk 15 feet, and kilocalories expended per week	No mention	(40)
MiRNA-185-3p	Plasma	↑	The expression level was associated with muscle weight loss, grip strength, self-reported exhaustion, time to walk 15 feet, and kilocalories expended per week	No mention	(40)
MiRNA-194-3p	Plasma	↑	The expression level was associated with muscle weight loss, grip strength, self-reported exhaustion, time to walk 15 feet, and kilocalories expended per week	No mention	(40)
MiRNA-203a-3p	Serum	↑	The expression level was associated with the psoas muscle mass index and intramuscular adipose tissue content	No mention	(1)
MiRNA-326	Plasma	↑	The expression level was associated with muscle weight loss, grip strength, self-reported exhaustion, time to walk 15 feet, and kilocalories expended per week	No mention	(40)
MiRNA-424-5p	Muscle	↑	The expression level was associated with the result of 3-m gait speed and 6-m timed up and go test	Pol I R1A and UBTF	(16)
MiRNA-532-5p	Plasma	↑	The expression level was associated with muscle weight loss, grip strength, self-reported exhaustion, time to walk 15 feet, and kilocalories expended per week	No mention	(40)
MiRNA-576-5p	Plasma	↑	The expression level was associated with muscle weight loss, grip strength, self-reported exhaustion, time to walk 15 feet, and kilocalories expended per week	No mention	(40)
MiRNA-760	Plasma	↑	The expression level was associated with muscle weight loss, grip strength, self-reported exhaustion, time to walk 15 feet, and kilocalories expended per week	No mention	(40)

*miRNA, microRNA; mRNA, messenger RNA; PFKFB3, 6-phosphofructo-2-kinase/fructose-2-,6-bisphosphatase 3; Pol I R1A, polymerase I receptor 1A; PRKAA1, protein kinase AMP-activated catalytic subunit alpha 1; Smad, mothers against decapentaplegic; TGF-βR2, transforming growth factor-β receptor 2; UBTF, upstream binding transcription factor; VEGFA, vascular endothelial growth factor A; ↑ means miRNA's upregulation in each samples*.

**Table 2:** Associations between miRNA expression levels and sarcopenia in rodent models.

**MiRNA**	**Sample**	**Expression change**	**Details**	**Target mRNA**	**References**
MiRNA-1-3p	Muscle	↑	The expression level was associated with the soleus muscle weight/tibia length index, time to peak twitch tension, maximum tetanic contraction and half relaxation time of twitch, maximum tetanic relaxation rate, and fatigue index	TRIM63 and FBXO32	(28)
MiRNA-29	Muscle	↑	The expression level was associated with extensor digitorum longus and soleus muscle weight losses	B-myb, IGF-1, and p85	(38)
MiRNA-29a-3p	Muscle	↑	The expression level was associated with the soleus muscle weight/tibia length index, time to peak twitch tension, maximum tetanic contraction and half relaxation time of twitch, maximum tetanic relaxation rate, and fatigue index	TRIM63 and FBXO32	(28)
MiRNA-29b-3p	Muscle	↓	The expression level was associated with the soleus muscle weight/tibia length index, time to peak twitch tension, maximum tetanic contraction and half relaxation time of twitch, maximum tetanic relaxation rate, and fatigue index	TRIM63 and FBXO32	(28)
MiRNA-98-5p	Muscle	↑	The expression level was associated with the size of muscle fibers	NGF	(5)
MiRNA-133a-3p	Muscle	↑	The expression level was associated with the soleus muscle weight/tibia length index, time to peak twitch tension, maximum tetanic contraction and half relaxation time of twitch, maximum tetanic relaxation rate, and fatigue index	TRIM63 and FBXO32	(28)
MiRNA-133b-3p	Muscle	↑	The expression level was associated with the soleus muscle weight/tibia length index, time to peak twitch tension, maximum tetanic contraction and half relaxation time of twitch, maximum tetanic relaxation rate, and fatigue index	TRIM63 and FBXO32	(28)
MiRNA-181a	Muscle	↓	The expression level was associated with the size of myotubes	Sirt1	(71)
MiRNA-434-3p	Muscle	↓	No mention	Elf5A1	(60)
MiRNA-455-3p	Muscle	↓	The expression level was associated with the size of myotubes	PITX1 and RXRB	(42)

*miRNA, microRNA; mRNA, messenger RNA; B-myb, myeloblastosis-related protein B; Elf5A1, eukaryotic translation initiation factor 5A1; Fbxo32, F-box protein 32; IGF-1, insulin-like growth factor-1; NGF, nerve growth factor; PITX1, paired-like homeodomain transcription factor 1; RXRB, retinoid X receptor-β; Sirt1, sirtuin 1; Trim63, tripartite motif containing 63 protein; ↓ means miRNA's downregulation in each samples; ↑ means miRNA's upregulation in each samples*.

**Table 3:** Effects of modulation of miRNA expression on frailty and sarcopenia in cells *in vitro* and rodent frailty and sarcopenia models *in vivo*.

**MiRNA**	**Tx**	**Rodent model and/or cells**	**Effects**	**Target mRNA**	**References**
MiRNA-181a	OE	Mouse cultured myotubes of differentiated myoblasts (C2C12 cells)	Inhibition of Sirt1 induced a decrease in the myotube diameter	Sirt1	(71)
MiRNA-203a-3p	OE	Human skeletal muscle cells	Inhibition of BIRC5 induced a decrease in the number of skeletal muscle cells	BIRC5	(1)
MiRNA-434-3p	OE	Mice myocytes	Inhibition of Elf5A1 protected myocytes from apoptosis	EIF5A1	(60)
MiRNA-455-3p	OE	Mouse cultured myotubes of differentiated myoblasts (C2C12 cells)	Inhibition of PITX1 and RXRB induced larger diameters of C2C12 myotubes	PITX1 and RXRB	(42)
MiRNA-672-5p	OE	Ovariectomy-induced sarcopenia mouse gastrocnemius muscle	Inhibition of Atrogin-1 and MuRF1 induced a decrease in lean muscle mass and Feret's diameter of muscle fibers and increase in the serum creatinine kinase level	Atrogin-1 and MuRF1	(6)

*miRNA, microRNA; mRNA, messenger RNA BIRC, baculoviral inhibitors of apoptosis repeat containing; Elf5A1, eukaryotic translation initiation factor 5A1; MuRF1, muscle ring-finger protein-1; OE, overexpression; PITX1, paired-like homeodomain transcription factor 1; RXRB, retinoid X receptor-β; Sirt1, sirtuin 1; Tx, treatment*.

### Changes of the Expression Levels of microRNAs in Sarcopenia

Expression of 13 miRNAs (miRNA-10a-3p,−19a,−21, 34a,−92a-3p, 185-3p, 194-3p,−203a-3p,−326,−424-5p,−532-5p,−576-5p, and−760) was found to be changed in the muscles and/or blood (serum or plasma) of patients with sarcopenia [Table 1; (1, 16, 40, 64, 82)]. Among them, expression of three miRNAs in muscle (miRNA-19a,−34a, and−424-5p,) (16, 82), eight miRNAs in plasma (miRNA-10a-3p,−92a-3p,−185-3p,−194-3p,−326,−532-5p,−576-5p, and−760) (40), and two miRNAs in serum (miRNA-21 and−203a-3p) (1, 64) was changed and associated with physical functions including shrinking, weakness, poor endurance and energy, slowness, and low physical activity levels and expression of many signaling molecules, such as protein kinase AMP-activated catalytic subunit alpha 1 (PRKAA1), 6-phosphofructo-2-kinase/fructose-2-,6-biphosphatase 3 (PFKFB3), transforming growth factor-β receptor 2 (TGF-βR2), vascular endothelial growth factor A (VEGFA), polymerase I receptor 1A (Pol I R1A), and upstream binding transcription factor (UBTF), which were shown to contribute to sarcopenia development [Table 1; (16, 64, 82)].

Expression of 10 miRNAs (miRNA-1-3p,−29,−29a-3p,−29b-3p,−98-5p,−133a-3p,−133b-3p,−181a,−434-3p, and−455-3p) was changed in muscles of rodent models of sarcopenia [Table 2; (5, 28, 38, 42, 60, 71)]. These miRNAs were associated with the expression levels of many signaling molecules, including tripartite motif containing 63 protein (TRIM63), F-box protein 32 (FBXO32), myeloblastosis-related protein B (B-myb), insulin-like growth factor-1 (IGF-1), p85, nerve growth factor (NGF), sirtuin 1 (Sirt1), eukaryotic translation initiation factor 5A1 (Elf5A1), paired-like homeodomain transcription factor 1 (PITX1), and retinoid X receptor-β (RXRB), which were shown to contribute to sarcopenia development [Table 2; (5, 28, 38, 42, 60, 71)].

### Effects of microRNA Modulation on Sarcopenia

**Figure 2:**
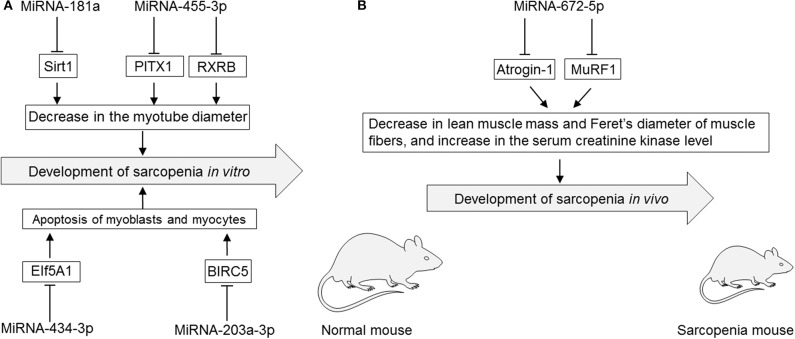
**(A)** Roles of miRNAs in cultured myotubes, myoblasts, and myocytes *in vitro*. **(B)** Roles of miRNAs in rodent sarcopenia models *in vivo*. EIf5A1, eukaryotic translation initiation factor 5A1; miRNA, microRNA; PITX1, paired-like homeodomain transcription factor 1; RXRB, retinoid X receptor-β; Sirt1, sirtuin 1; MuRF1, muscle ring-finger protein-1.

Several studies have reported that modulation of miRNAs has significant effects on sarcopenia in cultured myocytes *in vitro* (Table 3, Figures 2A,B). The specific miRNAs that have been reported to affect sarcopenia are described below.

### MiRNA-181a

MiRNA-181a binds to the 3′-untranslated region of Sirt1 that is implicated in influencing aging, apoptosis, and inflammation (71). Overexpression of miRNA-181a using an miRNA-181a mimic by lipofection was shown to significantly decrease the myotube diameter, which was mediated by inhibiting its target Sirt1 in cultured myotubes of differentiated C2C12 cells, a subclone of mouse myoblasts. However, miRNA-181a knockdown using a miRNA-181a inhibitor led to an increase in the myotube diameter of cultured myotubes of differentiated C2C12 cells (71).

### MiRNA-203a-3p

MiRNA-203a-3p binds to the 3′-untranslated region of baculoviral inhibitors of apoptosis repeat containing 5 (BIRC5), a member of the apoptosis inhibitor protein family that suppresses apoptosis via inhibition of the initiator caspase 9 and executers caspase 3 and 7 (84). MiRNA-203a-3p was upregulated in serum of colorectal cancer patients with sarcopenia as evaluated by a lower psoas muscle mass index compared with than in colorectal cancer patients without sarcopenia (1). Knockdown of miRNA-203a-3p using an miRNA-203-3p mimic by lipofection inhibited cell proliferation and induced apoptosis via increasing the expression level of the target BIRC5 in cultured human skeletal muscle cells (1).

### MiRNA-434-3p

MiRNA-434-3p binds to the 3′-untranslated region of EIf5A1 that is involved in many cellular processes including cell division, apoptosis, and inflammation (60). Overexpression of miRNA-434-3p using an miRNA-434-3p mimic by lipofection inhibited the expression levels of EIf5A1, which prevented apoptosis of apoptosis-stimulated primary myocytes purified from hind limb muscles of C57BL/6J mice (60).

### MiRNA-455-3p

Overexpression of miRNA-455-3p using an miRNA-455-3p mimic by lipofection inhibited the expression levels of PITX1 and RXRB, which are involved in muscle dystrophy and aging, resulting in a significant increase of the diameter of cultured myotubes differentiated from cultured mouse C2C12 myoblasts (42).

### MiRNA-672-5p

Overexpression of miRNA-672-5p via tail vein injection of an miRNA-672-5p mimic in liposomes alleviated ovariectomy-induced sarcopenia in female BALB/c mice (6). Overexpression of miRNA-672-5p in ovariectomy-induced sarcopenia mice increased lean muscle mass but decreased the serum creatinine kinase level and increased Feret's diameter of muscle fibers with inhibited muscle atrogenes (Atrogin-1 and Murif1) that stimulate protein catabolism and negatively affect muscular health (6). Overexpression of miRNA-672-5p in ovariectomy-induced sarcopenia mice also increased osteoblastogenesis and mineralization, thereby reversing bone loss (6).

## Discussion

Many miRNAs increase or decrease in muscles and blood of patients with sarcopenia and rodent models of sarcopenia. These expression changes are associated with the phenotypes of sarcopenia, such as lower physical functions and expression levels of many signaling molecules that mediate progression of sarcopenia. These lines of evidence suggest that miRNA levels in muscles and/or blood are potential biomarkers for sarcopenia. However, no study has reported the relationship between the expression changes of plasma/serum and muscle miRNAs in sarcopenia. It is necessary to investigate this relationship to clarify the mechanisms of miRNAs in each organ including muscles and their circulation form in blood for the development of sarcopenia as well as the utility of miRNAs in blood as biomarkers of sarcopenia. Additionally, several miRNAs have been demonstrated to affect sarcopenia in myocytes *in vitro* or rodent sarcopenia models *in vivo*. All studies reported the effects of miRNAs on sarcopenia in the setting of overexpression of these miRNAs as shown in Table 3. These results suggest that miRNAs are potential targets of gene therapy for sarcopenia. However, further studies are needed to investigate the mechanisms, target cells, and adverse effects of modulating these miRNAs. Additionally, so far, there is no clinical study that has directly investigated the functions of miRNAs in sarcopenia by modulation of their expression using a mimic and/or inhibitor. Future clinical studies will be necessary to confirm the effects of miRNAs on sarcopenia and their potential targets for gene therapy of sarcopenia. Our review has a number of limitations. First, we only searched for studies published in English. Second, we only used the PubMed database to identify publications. Third, meta-analysis could not be performed because the number of studies reporting miRNAs for sarcopenia was small, so statistical power would have been low. Therefore, further research is warranted to verify our conclusions.

## Conclusion

Many miRNAs increase or decrease in muscles and blood of patients with sarcopenia and rodent models of sarcopenia. Additionally, several miRNAs have been demonstrated to affect sarcopenia in myocytes *in vitro* or rodent sarcopenia models *in vivo*. These results suggest that miRNAs are potential biomarkers and targets of gene therapy for sarcopenia. Further studies including clinical studies will be necessary to confirm the utility of miRNAs as biomarkers and targets for gene therapy of sarcopenia.

## Author Contributions

All authors listed have made a substantial, direct and intellectual contribution to the work, and approved it for publication.

## Conflict of Interest

The authors declare that the research was conducted in the absence of any commercial or financial relationships that could be construed as a potential conflict of interest.
